# Transcriptomic profiling reveals immune signatures associated with potential COVID-19 susceptibility and a predictive framework in a Chinese cohort

**DOI:** 10.3389/fimmu.2026.1847360

**Published:** 2026-08-21

**Authors:** Yuting Xin, Chao Yi, Weiyu Zhu, Jie Zhang, Dongli Wang, Kai Zhuang, Jian Chen, Peiwen Cheng, Jingru Feng, Qiuhan Lu, Wenjie Han, Hao Zheng, Jing Tang, Tieqiang Wang, Xiangjun Du

**Affiliations:** 1 School of Public Health (Shenzhen), Sun Yat-sen University, Guangzhou, China; 2 School of Public Health (Shenzhen), Shenzhen Campus of Sun Yat-sen University, Shenzhen, China; 3 Guang Ming District Center for Disease Control and Prevention, Shenzhen, China; 4 Bao’an District Hospital for Chronic Diseases Prevention and Cure, Shenzhen, China; 5 Shenzhen Key Laboratory of Pathogenic Microbes & Biosafety, Shenzhen Campus of Sun Yat-sen University, Shenzhen, China; 6 Key Laboratory of Tropical Disease Control, Ministry of Education, Sun Yat-sen University, Guangzhou, China

**Keywords:** COVID-19, immune response, molecular pathways, prediction model, susceptibility

## Abstract

**Introduction:**

Despite significant interindividual susceptibility to COVID-19, the molecular basis of host vulnerability in the Chinese population remains poorly defined.

**Methods:**

Leveraging the large-scale Omicron exposure following China’s transition from “zero-COVID,” we conducted transcriptomic profiling of peripheral blood mononuclear cells (PBMCs) from 173 volunteers stratified into high susceptibility (HS) and low susceptibility (LS) groups based on SARS-CoV-2-specific antibody levels and retrospective symptom assessments. Differential expression analysis, protein-protein interaction (PPI) network analysis, weighted gene co-expression network analysis (WGCNA), and single-sample gene set enrichment analysis (ssGSEA) were performed. An ensemble machine-learning model was trained on 80% of the cohort and evaluated on the remaining 20% test set.

**Results:**

Differentially expressed genes were enriched in neutrophil recruitment and T cell activation, while PPI analysis prioritized hub genes involved in inflammation, chemotaxis, and endothelial integrity. WGCNA identified two modules negatively correlated with high susceptibility, enriched in MHC class II antigen presentation and T cell activation/epigenetic regulation, respectively. HS individuals showed enrichment of interferon-related transcriptional signatures, together with reduced expression of interferon receptor-associated genes and suppression of B-cell receptor and complement pathways. The ensemble model achieved an AUC of 0.92 on the independent test set.

**Discussion:**

These findings identify transcriptomic signatures associated with potential COVID-19 susceptibility across innate, adaptive, and vascular immune-related axes and provide an exploratory framework for risk classification and potential precision prevention.

## Introduction

SARS-CoV-2, a highly contagious β-coronavirus, triggered a global pandemic marked by unprecedented transmission rates and clinical variability (1). Despite the transition to a post-pandemic era, COVID-19 continues to pose a persistent challenge to global public health. Notably, similar viral exposure results in divergent outcomes: ranging from asymptomatic carriage (32% of Omicron-positive cases (2)) to life-threatening pneumonia, highlighting inherent host susceptibility differences determining symptomatic infection likelihood. Elucidating the mechanisms is critical for advancing precision public health strategies to mitigate ongoing COVID-19 burden and prepare for future pandemic threats.

Host susceptibility is a multifactorial trait influenced by genetic background (3), age (4), sex (5), and nutritional status (6), with innate and adaptive immune responses playing pivotal roles in resisting viral invasion and shaping disease progression (7). Accumulating evidence indicates that pre-infection expression levels of specific genes and profiles of circulating immune cells collectively form “immunological set points” that determine host defense efficacy prior to pathogen exposure, while post-exposure responses act as modulators that amplify or constrain these pre-existing reserves. These baseline immune features not only underpin core host defense mechanisms but also correlate closely with infection outcomes, holding promise as predictive biomarkers for disease trajectories. For instance, asymptomatic H3N2 carriers demonstrated elevated baseline expression related to “defense response to the virus” and “activation of immune response” at baseline compared to symptomatic patients (8). A recent study found that the neutrophil-to-lymphocyte ratio(NLR) independently predicts influenza susceptibility (9). Longitudinal data further reveal that baseline innate and T cell populations can improve the prediction of symptomatic influenza susceptibility over vaccination, demographics or serology alone (10). For respiratory syncytial virus (RSV) infection, a study found that the regulation of neutrophils, peptidases, and IL-17-related pathways may affect antiviral protection and the activation of mucosal neutrophils can highly predict symptomatic before virus exposure (11). This paradigm extends to COVID-19: human SARS-CoV-2 challenge studies reveal that pre-exposure overexpression of HLA-DQA2 in nasopharyngeal and blood cells is associated with SARS-CoV-2 resistance, whereas rapid early innate immune responses (e.g., MAIT cells) and regulatory T cell-mediated inflammation control jointly determine infection outcomes (12). Furthermore, recent evidence suggests that “functional protection” against primary infection is mediated by heightened baseline levels of nasopharyngeal CCL13, which is locally produced by conventional dendritic cells and monocytes, as well as the presence of cross-reactive T cells and less differentiated NK cells that promote rapid innate-like responses to effectively block the infection (13). These findings suggest that host defense efficacy is predominantly shaped by immunological set points established prior to pathogen exposure, with post-exposure responses functioning as modulators that amplify or constrain pre-existing functional reserves. This framework highlights the underappreciated predictive value of baseline immune states in clinical trajectories.

While controlled human infection models have advanced susceptibility research, their utility is often hindered by ethical barriers, limited scalability, and inability to capture population-level immunological heterogeneity-particularly under divergent pandemic policies (14, 15). Furthermore, systematic transcriptomic investigations into the underlying molecular mechanisms, especially at the transcriptome level, driving COVID-19 susceptibility specifically within the Chinese population remains limited. Existing research has predominantly focused on identifying susceptibility-associated genetic loci (e.g.,through genome-wide association studies (16, 17)). Single-cell transcriptomic studies have provided important insights into immune dysregulation in COVID-19, particularly highlighting alterations in monocyte function, antigen presentation (MHC class II), Toll-like receptor signaling, and interferon responses (18, 19). However, comprehensive transcriptomic profiling related to COVID-19 susceptibility from the perspective of functional mechanism and predictive model are notably lacking, which is also different from the post-infection signatures. China’s abrupt transition from zero-COVID containment to rapid surge mitigation created an unparalleled natural experiment: demographically diverse populations experienced homogeneous viral exposure to the Omicron variants (after-lifting COVID-19 wave), effectively isolating host-intrinsic susceptibility factors from environmental confounders (20, 21). This epidemiological singularity, combined with the standardized contact networks of university communities (22), provides a quasi-controlled platform to decipher susceptibility mechanisms.

Recognizing the potential of this unique epidemiological situation, we designed a comprehensive study to investigate COVID-19 susceptibility mechanism. We carefully selected 173 volunteers from a university-based population. Although pre-exposure samples would most directly reflect intrinsic susceptibility, PBMCs collected more than three months after the Omicron wave were used here as an approximation of a stable post-recovery immune state. Here, we examined a potential susceptibility phenotype defined by combined clinical and serological responses during the after-lifting COVID-19 wave in a Chinese cohort. By conducting serological antibody profiling and symptom-based retrospective analysis, we were able to accurately determine their susceptibility levels. This initial step was crucial as it provided a reliable basis for classifying individuals into different susceptibility groups. Subsequently, we isolated peripheral blood mononuclear cells (PBMCs) from the volunteers for transcriptomic sequencing. This was followed by a series of in-depth analyses, including differential expression analysis, PPI, WGCNA and ssGSEA to identify susceptibility-associated genes and key biological pathways. An exploratory ensemble machine-learning model was then constructed by integrating these biomarker sets and showed acceptable predictive performance on the held-out test set. Thus, the novelty of this study lies in linking multidimensional PBMC transcriptomic characterization with a preliminary predictive framework for potential COVID-19 susceptibility in a Chinese cohort.

## Methods

### Recruitment and categorization of volunteers

Following the relaxation of China’s zero-COVID policy in late 2022, a large-scale Omicron-related infection wave (after-lifting COVID-19 wave) emerged nationwide, providing a unique epidemiological setting for population-level exposure, 173 volunteers were recruited and asked to fill out a retrospective questionnaire detailing self-reported symptoms related to the after-lifting COVID-19 wave between December 2022 and January 2023(**Table 1**). We assumed that transcriptomic profiles would have largely returned to baseline levels more than three months after SARS-CoV-2 infection in healthy young individuals (23–25). From April 2023, peripheral blood samples (10 mL per individual) were collected in serum separator tubes for serum preparation and in acid citrate dextrose (ACD) or ethylenediaminetetraacetic acid (EDTA) anticoagulant tubes for PBMC and plasma isolation. In the present study, potential COVID-19 susceptibility was operationally defined as the relative tendency to develop clinically apparent infection during the after-lifting COVID-19 wave, based on the combined assessment of symptom status and SARS-CoV-2-specific IgG levels. The 173 samples were classified into two groups: a) The high susceptibility (HS) group (70 cases) includes participants whose IgG antibody level ≥100 (AU/mL) with symptoms during the after-lifting COVID-19 wave. Previous studies indicate that symptomatic SARS-CoV-2 infection is generally associated with higher antibody levels; therefore, these participants were operationally classified as having a higher likelihood of clinically apparent infection (26). b) The low susceptibility (LS) group (103 cases) includes participants whose IgG antibody level <100 (AU/mL) or who are asymptomatic during the after-lifting COVID-19 wave. These participants were operationally assigned to the LS group based on either a lower SARS-CoV-2-specific IgG level or the absence of reported symptoms during the after-lifting COVID-19 wave. In other words, the LS group comprises individuals who either with IgG antibody level<100(AU/ml) (with or without symptoms) or experienced asymptomatic infection during the after-lifting COVID-19 wave, a standard grouping method used by other studies that distinguishes strength difference from the perspective of host response (9). These criteria aimed to isolate natural infection-related susceptibility from vaccine-mediated protection. Inclusion criteria included: a) History of good physical health, with no chronic respiratory disease, malignancy, cardiovascular or cerebrovascular disease, metabolic disorder, blood disorder, autoimmune disease, or immunosuppressive or immunodeficient disease. No history of HIV, hepatitis B or C infections, pregnancy, lactation, or long-term medication use. b) No history of smoking. c) No recent vaccinations within the past three years (excluding COVID-19 vaccines), and no diagnosed infectious diseases (excluding COVID-19 infection).

**Table 1:** Baseline characteristics of participants.

Characteristics	LS group (n=103)	HS group (n=70)	*P* value
Age	20(19-21)	20(20-22)	0.301
BMI	21(20-23)	21(20-23)	0.476
Gender			0.633
Female	45(43.7%)	34(48.6%)	
Male	58(56.3%)	36(51.4%)	
Symptoms scores			<0.001
Cough	0	3.5(1-6.8)	<0.001
Fatigue	0	5(3-7)	<0.001
Sore throat	0	5(2-7)	<0.001
Anosmia/ageusia	0	1(0-4)	<0.001
Nasal obstruction	0	4(1-6)	<0.001
Runny nose	0	4(1-5)	<0.001
conjunctivitis	0	0	<0.001
Muscle soreness	0	3.5(1-6)	<0.001
Diarrhea	0	0	<0.001
Total scores	0	27.5(18-38.5)	<0.001
Post-vaccination interval	447(379-513.5)	488(425.8-533)	0.077
Booster dose administration status			0.623
Yes	73(70.9%)	52(74.3%)	
No	30(29.1%)	18(25.7%)	
laboratory parameters			
IgG(AU/mL )	154.4(42.4-369.1)	536.5(419.7-661.1)	<0.001
IgM(AU/mL )	0.4(0.3-0.6)	0.4(0.3-0.6)	0.526

Values are presented as median (IQR) or n (%). BMI, body mass index; IQR, interquartile range.

### Serological assessment anti-SARS-CoV-2 antibody levels

Serum samples were assayed to determine the concentration of anti-SARS-CoV-2 IgG using an iFlash 3000-H chemiluminescence immunoassay analyser (Shenzhen YHLO Biotech, Shenzhen, China) with an iFlash-SARS-CoV-2 IgG kit and iFlash-SARS-CoV-2 IgM kit (27).

### PBMC isolation, RNA extraction, library construction

Peripheral blood mononuclear cell (PBMC)s were obtained by Ficoll-Paque density centrifugation and stored at -80 °C with serum-free cell freezing solution immediately after isolation and transferred to liquid nitrogen after about 24 h for storage. Total RNA was isolated and purified using TRIzol following the manufacturer’s procedure. mRNA sequencing libraries were constructed using the Optimal Dual-mode mRNA Library Prep Kit (BGI-Shenzhen, China). Polyadenylated mRNA was enriched via oligo(dT) magnetic beads, fragmented enzymatically, and reverse-transcribed into double-stranded cDNA. After end repair, A-tailing, and adaptor ligation, libraries were PCR-amplified and quality-controlled. Single-stranded circular DNA was generated through denaturation and splint ligation, followed by rolling circle amplification with phi29 polymerase to produce DNA nanoballs (DNBs >300 copies).

### RNA sequencing, mapping and quantifications

Sequencing was performed on the BGISEQ-500 platform (PE150 mode) using patterned nanoarrays. SOAPnuke was used for integrated quality control and preprocessing (28). Reads that passed all quality control steps were then aligned to the human genome (GRCh38 release110) by using HISAT2 (29)(v2.2.1) and quantified by using Subread package (30) (v.1.5.2). 15,580 genes among 62,754 expressed genes were selected and then normalized to transcripts per million (TPM) for further analysis (only protein-coding genes and normalized count >0 in at least 50% of samples were kept). Differential expression analysis between the HS and LS groups was performed using DESeq2 based on raw gene-level read counts. The design formula included susceptibility group as the explanatory variable, with the LS group as the reference. Age, sex, BMI, post-vaccination interval, and booster vaccination status were comparable between the two groups and were therefore not included as additional covariates (31). The cutoff *p* value was 0.05, and the cutoff |log2FC| value was 0.25.

### Gene function annotation

Transcriptomic GO function enrichment, BTM pathways enrichment were performed using the clusterProfiler (32). Blood transcriptional module (BTM) analysis was carried using a pre-defined set of modules which is a systems immunology framework constructed through integrative analysis of >30,000 human blood transcriptomes across 500 studies to captured distinct immunological processes (33). Additionally, the visualization of the enrichment results is implemented by using the R package aPEAR (34).

### Protein-protein interaction network and identification of common feature genes

PPI of 571 DEGs between the HS and LS groups were analyzed by STRING (35) (https://cn.string-db.org), and the PPIs with a score > 0.4 were visualized as a network using Cytoscape (v3.10.2), The top 30 nodes (genes) were scored and networked with degree by CytoHubba (36) and annotated by stringApp (37).

### Weighted gene co-expression network analysis

To delineate co-expression patterns associated with susceptibility trait, genes were ranked by median absolute deviation (MAD) to select the top 3,000 most variable transcripts for co-expression network construction using the WGCNA R package (38). Samples were initially clustered hierarchically using the flashClust algorithm to detect potential outliers (**Supplementary Figure 4A**). The optimal soft-thresholding power was determined using the pickSoftThreshold function by jointly considering the scale-free topology fit index and mean connectivity (**Supplementary Figures 4B, C**). A signed hybrid co-expression network was subsequently generated using the blockwiseModules function, with transcripts partitioned into distinct modules through dynamic tree-cutting (maxBlockSize = 6000, TOMType = “unsigned”, minimum module size = 50 genes).

Module-trait associations were analyzed by computing module eigengenes (MEs), defined as the first principal components capturing maximal expression variance within each module. Clinical metadata, including COVID-19 susceptibility, were numerically encoded to enable linear regression modeling between MEs and phenotypic traits. Then module-trait relationships analysis was performed. The p-value was calculated by the function “corPvalueStudent” in the R package of WGCNA. And the significance of modules was determined with p ≤ 0.05. Consequently, genes in these modules were applied functional annotation.

Hub genes associated with susceptibility were prioritized by quantifying intramodular connectivity through two complementary metrics: gene significance (GS), which reflects the strength of association between individual gene expression and clinical phenotypes, and module membership (MM), which represents the correlation of gene expression profiles with module eigengenes.

### Evaluation of immune infiltration

The infiltration levels of the activity of 24 immune-related pathways (39) were systematically evaluated using single-sample gene set enrichment analysis (ssGSEA). Using the ‘GSVA’ R package (40), this framework quantified PBMC immune features at single-sample resolution, with ssGSEA scores representing pathway activity states.

### Machine learning method for COVID-19 susceptibility prediction model

To construct an integrated susceptibility prediction model, we first divided the full dataset (n = 173) into a training cohort (80%) and an independent test cohort (20%), ensuring balanced distributions of susceptibility labels across sets. All subsequent analyses including feature selection and model training were performed exclusively within the training cohort. Recursive feature elimination with cross-validation (RFECV) was then applied separately to three biomarker sources (30 PPI hub genes, 68 WGCNA-derived module genes, and 312 ssGSEA-identified immune pathway genes), yielding 10 optimal features from each set (**Supplementary Table 4**).

For classifier selection, we benchmarked a panel of candidate algorithms tailored to each biomarker set. Specifically, random forest (RF) was evaluated for PPI and WGCNA-derived genes, whereas logistic regression (LR) was considered for ssGSEA-derived immune pathway genes. Hyperparameters for all models were optimized using an Optuna-based Bayesian search. For each biomarker set, the algorithm achieving the best performance in the training cohort was retained as the baseline classifier: RF (PPI), RF (WGCNA), and LR (ssGSEA) (**Supplementary Table 3**).

Model hyperparameters were optimized within the training cohort using nested 5-fold cross-validation, and predictive performance was then assessed on the independent test set using accuracy, precision, recall, F1-score, and AUC (**Table 2**; **Supplementary Table 5**). Finally, we implemented a two-layer stacking ensemble: probability scores from the three base models were concatenated as input features and used to train a logistic regression meta-learner. This stacking framework achieved superior generalizability with an AUC of 0.92 on the test set, confirming the robustness of cross-omics integration.

**Table 2:** Performance of different classifiers to predict COVID-19 susceptibility on the testing dataset.

Classifier	Accuracy	Precision	Recall	F1 score	AUC
RF (PPI)	0.77	0.70	0.58	0.64	0.85
RF (WGCNA)	0.77	0.67	0.67	0.67	0.85
LR (ssGSEA)	0.69	0.55	0.50	0.52	0.78
Ensemble Model	0.86	0.82	0.75	0.78	0.92

### Statistical and visualization analysis

All statistical analyses were performed using R software (version 4.4.2). Quantitative data were shown as the median with IQR. The statistical analyses were performed using the unpaired, two-sided Mann-Whitney U test. For all statistical analyses, *p* < 0.05 was considered statistically significant. Cowplot, aPEAR, ggrepel, gridExtra, ggplot2 were used for visualization purposes.

## Results

### Overview of the study participants

Based on a group of healthy young people who reported no post-COVID-19 syndrome, we tried to explore the COVID-19 susceptibility based on transcriptome data after more than three months of infection as an indicator of baseline status (23–25). To characterize the immunological mechanisms underlying COVID-19 susceptibility, we recruited 173 college student volunteers who experiencing the COVID-19 wave (December 2022-January 2023) following the lifting of China’s zero-COVID policy (the after-lifting COVID-19 wave). PBMCs were isolated from these participants, and transcriptomic sequencing was performed to explore molecular signatures associated with susceptibility. These participants were stratified into low susceptibility group (LS, IgG antibody level < 100 (AU/mL) or who are asymptomatic during the after-lifting COVID-19 wave) and high susceptibility (HS, IgG antibody level ≥ 100 (AU/mL) with symptoms during the after-lifting COVID-19 wave) based on symptom severity scores and IgG seropositivity threshold (**Table 1**). These two groups demonstrated balanced demographic and vaccination-related profiles, with no significant differences in age, BMI, sex distribution, post-vaccination intervals, and booster dose administration status. Despite this comparability, the HS group exhibited markedly higher frequencies of COVID-19-associated symptoms (including cough, fatigue, etc.) and elevated anti-spike IgG levels, whereas IgM titers showed no intergroup disparity.

### Pathway enrichment of DEGs and identification of hub genes

**Figure 1:**
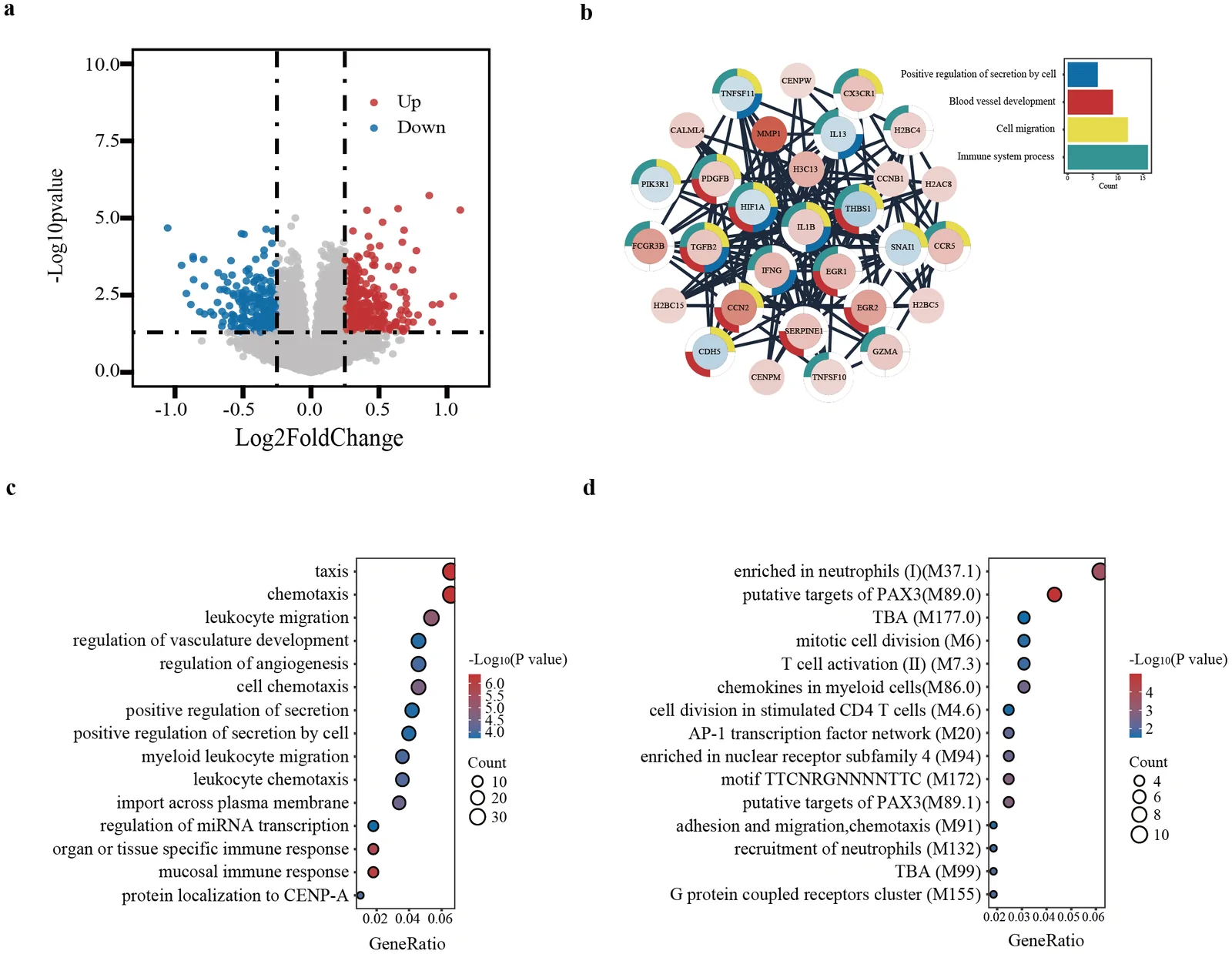
Differentially expressed genes analysis and pathway enrichment. **(A)** Volcano plot of DEGs identified between the HS and LS groups (thresholds: |Log_2_FC| > 0.25, *p*-value < 0.05; red: upregulated in HS, blue: downregulated). **(B)** CytoHubba-derived hub genes. Top 30 nodes ranked by degree centrality, with labels indicating key functional categories. nodes colored by Log_2_FC (red: up, blue: down). **(C)** Bubble chart displaying the top 15 GO: BP terms enriched for DEGs. **(D)** Bubble chart displaying the top 15 BTM gene sets enriched for DEGs.

To explore genes related to human susceptibility to SARS-CoV-2 at baseline, we performed comparative PBMC transcriptomic profiling between high and low susceptibility groups. Differential expression analysis (|log_2_FC| ≥ 0.25, *p* < 0.05) identified 571 DEGs, including 341 upregulated and 230 downregulated genes in HS individuals (**Figure 1A**). Gene Ontology Biological Process (GO: BP) analysis revealed significant enrichment in “cell chemotaxis”, “positive regulation of cell secretion”, “angiogenesis regulation”, and “mucosal immune response” (**Figure 1C**; **Supplementary Figure 2**). Given that our transcriptomic data were derived from PBMCs, we further applied blood transcriptional modules (BTMs) analysis. BTMs is a blood-specific systems immunology framework which can better capture circulating immune processes (33). The results showed significant enrichment in “neutrophil recruitment (M132)” and “T cell activation (M7.3)” modules (**Figure 1D**), highlighting myeloid and lymphoid compartment involvement in susceptibility phenotypes.

To identify functionally pivotal regulators underlying COVID-19 susceptibility, we first constructed a protein-protein interaction (PPI) network using high-confidence interactions (score > 0.4) from the STRING database (**Supplementary Table 1**). We performed centrality analysis via the CytoHubba plugin to prioritize hub genes based on degree centrality, identifying 30 key regulators that emerged as critical nodes in the network (**Figure 1B**; **Supplementary Table 1**). These hub genes spanned multiple functional categories: pro-inflammatory mediators (IL1B, IFNG), which drive cytokine storm responses; chemotaxis regulators (CCR5, CX3CR1), which mediate immune cell recruitment; hypoxia-fibrosis drivers (SERPINE1, HIF1A), which link oxygen stress to tissue remodeling; and endothelial barrier destabilizers (CDH5), which compromise vascular integrity. Together, these genes delineate a molecular landscape of immune-endothelial crosstalk that influences COVID-19 susceptibility, as further supported by functional enrichment analyses of related pathways.

### Identification of gene co-expression modules and hub gene extraction

**Figure 2:**
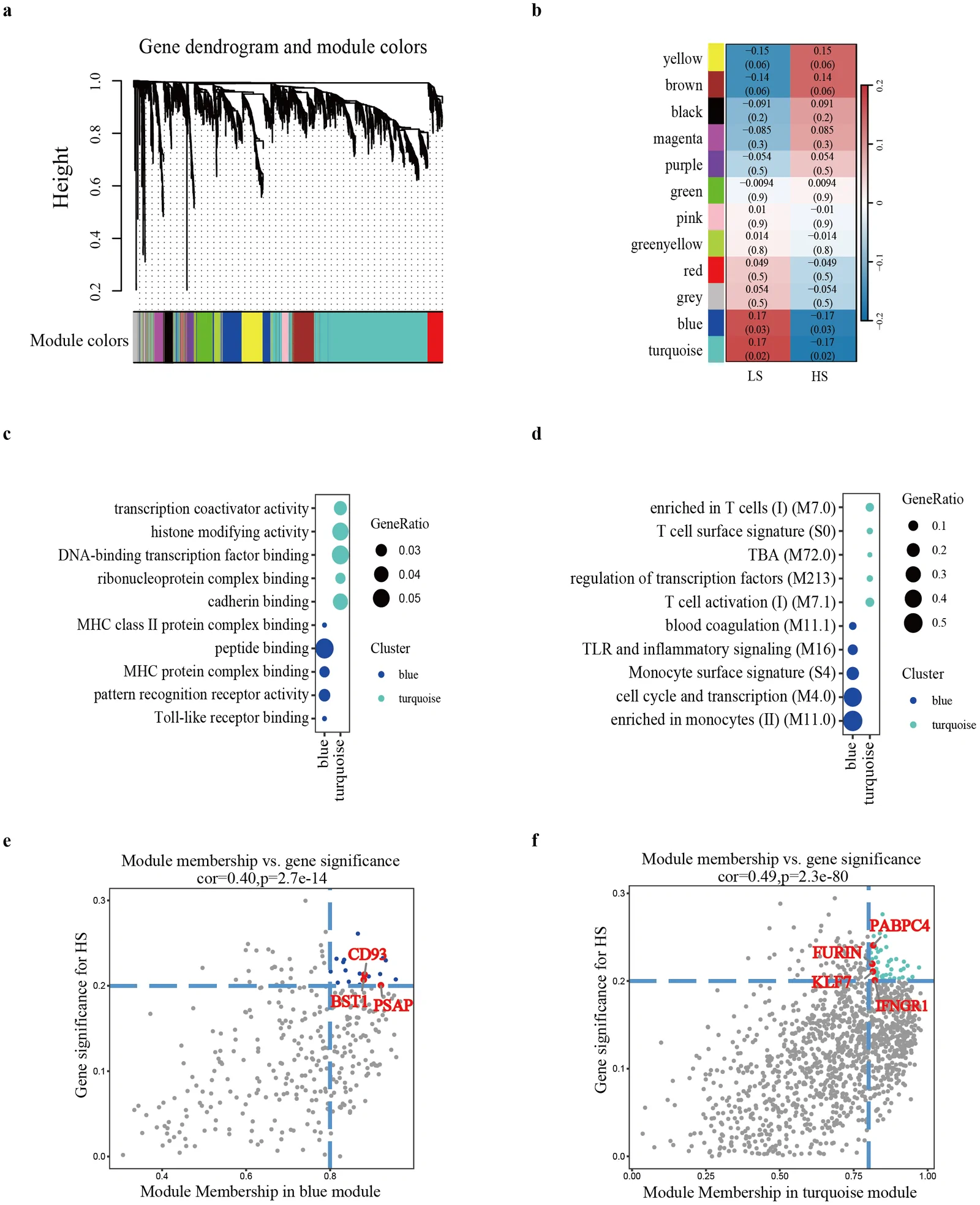
Co-expression network analysis by WGCNA. **(A)** Hierarchical clustering dendrogram with module assignments. Branches represent gene clusters with high topological overlap, and colors denote distinct co-expression modules. **(B)** Module-trait correlation matrix showing Pearson coefficients (with p-values) between module eigengenes (rows) and susceptibility phenotypes (columns). **(C)** GO: BP enrichment analysis of genes in the blue and turquoise modules. **(D)** BTM terms enrichment analysis of genes in the blue and turquoise modules. **(E)** Hub gene identification in the blue module, and the blue dots in the first quadrant represent hub genes. **(F)** Hub gene identification in the turquoise module, and the turquoise dots in the first quadrant represent hub genes.

While prior DEGs and PPI analyses revealed susceptibility-associated pathways and genes, host susceptibility arises from coordinated polygenic networks rather than isolated gene effects. ​To systemically decode this complexity, we applied weighted gene co-expression network analysis (WGCNA), identifying transcriptome-wide modules that mechanistically link single-gene findings to network-level pathophysiology across susceptibility strata. Weighted gene co-expression network analysis delineated 12 transcriptionally coordinated modules, with the gray module (containing unassigned genes) excluded from downstream analysis (**Figure 2A**). To identify susceptibility-associated molecular networks, we performed module-trait correlation analysis, which revealed two modules significantly associated with susceptibility: the blue module (r = -0.17, *p* = 0.03) and turquoise module (r= -0.17, *p* = 0.02), both exhibiting negative correlations with high susceptibility (**Figure 2B**). Notably, the inverse correlation coefficients suggested that higher expression of these modules’ genes corresponds to reduced disease vulnerability.

To characterize the biological significance of these modules, we performed integrative functional annotation. GO: BP analysis revealed that the blue module was significantly enriched in antigen presentation pathways, including “MHC class II binding” and “Toll-like receptor binding” (**Figure 2C**). Conversely, the turquoise module showed enrichment in epigenetic regulation processes, such as “transcription coactivator activity” and “histone modification” (**Figure 2D**). Blood Transcriptional Module (BTM) analysis further demonstrated that the blue module was associated with “monocyte surface signature (S4)”, whereas the turquoise module showed enrichment in “T cell activation (I) (M7.1)” (**Figure 2D**). Collectively, these results highlight a coordinated dysregulation of T cell epigenetic networks and monocyte-driven inflammatory responses as critical determinants of COVID-19 susceptibility. To prioritize candidate regulators mediating these pathways, we applied stringent thresholds (gene significance > 0.2; module membership > 0.8), identifying 18 hub genes in the blue module (e.g., PSAP, CD93, BST1) and 50 in the turquoise module (e.g., FURIN, IFNGR1, KLF7, PABPC4).

### Difference of immune cell abundance and immune responses

**Figure 3:**
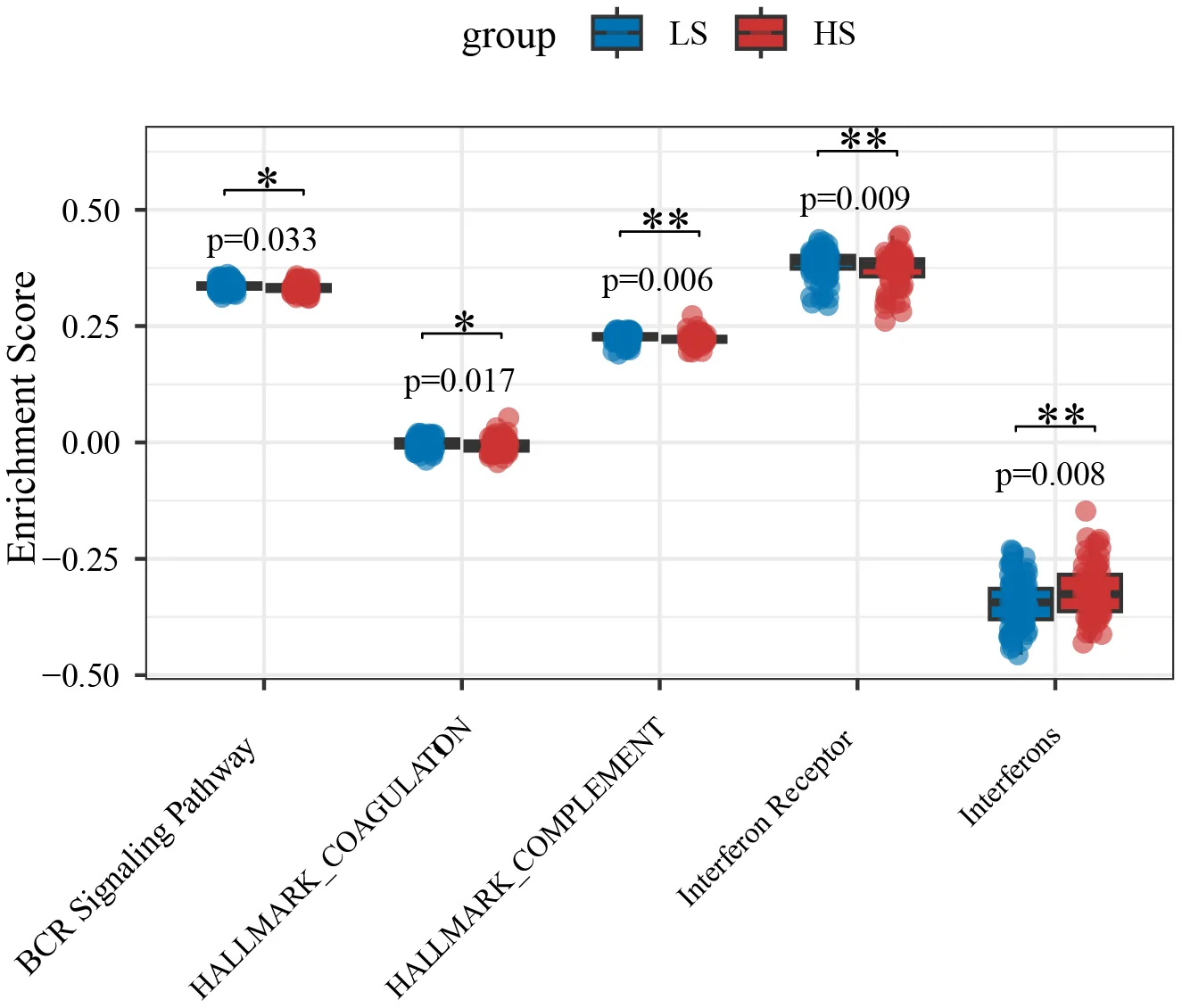
Comparison of immune cell infiltration and immune-related gene sets activity. Unpaired, two-sided Mann-Whitney U test p values are depicted in the plots, and the significant p-value cutoff was set at 0.05. The significant level was indicated by number of asterisks (* for p ≤0.05, ** for p ≤ 0.01, and *** for p ≤ 0.001).

Building on insights from differential expression and co-expression network analyses, both lines of evidence converged to implicate host immune status as a key determinant of COVID-19 susceptibility. To delineate immune landscape heterogeneity, we conducted single-sample gene set enrichment analysis (ssGSEA) utilizing 24 immune-related pathways derived from the C7 immunologic signature collection and hallmark gene sets in MSigDB (**Supplementary Table 5**). Comparative analysis revealed distinct immune dysregulation patterns in high susceptibility individuals. Specifically, these individuals displayed enrichment of interferon-related transcriptional signatures. Conversely, critical immune regulatory pathways including interferon receptor signaling, complement cascade activation, coagulation regulation, and B cell receptor (BCR) signaling were significantly suppressed in the HS group (**Figure 3**).

### Machine learning model building and characterization

To construct a predictive model of COVID-19 susceptibility, we integrated three biomarker sources: 30 PPI hub genes, 68 WGCNA module genes, and 312 ssGSEA-derived immune pathway features. The dataset (n = 173) was randomly divided into a training set (80%) and a test set (20%) with matched susceptibility distribution. Within the training cohort, recursive feature elimination with cross-validation (RFECV) was applied to each biomarker set, selecting 10 optimal features per category (**Supplementary Table 4**). Using these features, we developed three baseline classifiers: a random forest (RF) for PPI hub genes, a random forest (RF) for WGCNA module genes, and a logistic regression (LR) for ssGSEA immune pathways (**Supplementary Table 3**). When evaluated on the independent test set, the three models achieved AUC values of 0.85, 0.85, and 0.78 respectively (**Table 2**). To enhance predictive accuracy, the probability outputs of the three models were integrated into a logistic regression ensemble, which achieved an improved AUC of 0.92. Performance metrics of all models in both the training and test sets are summarized in **Supplementary Tables 5**, **3**, and the ensemble model achieved AUC = 0.98 in the training set but AUC = 0.92 in the independent test set, indicating strong but not overfitted performance.

## Discussion

The host immune response to SARS-CoV-2 is characterized by a remarkable degree of complexity, necessitating a delicate orchestration between innate signaling, adaptive cellular recruitment, and humoral memory. Although clinical outcomes vary significantly across individuals, the underlying immunological determinants of this susceptibility, especially in the context of a largely immunologically naive population, remain elusive. In this study, we addressed this gap by integrating multi-dimensional transcriptomic profiling of PBMCs with ensemble machine learning. By characterizing the molecular signatures, we have not only identified key regulatory hubs associated with host vulnerability but also provided an exploratory framework for the prospective assessment of infection risk.

Our differential gene expression and weighted co-expression network analyses consistently identified transcriptional patterns related to neutrophil recruitment and interferon signaling, together with adaptive immune processes involving T-cell activation and MHC-mediated antigen presentation. Within the PPI network, IL1B and CCR5 emerged as highly connected candidate genes associated with the HS phenotype. IL1B is known to participate in inflammasome signaling, NF-κB activation, neutrophil responses, neutrophil extracellular trap formation, and pro-inflammatory cytokine production (41, 42). This process leads to the excessive recruitment of immune cells, a mechanism highly consistent with the widely reported cytokine storm phenotype in severe COVID-19 patients (43). CCR5, a critical coreceptor for HIV infection (44), is also characterized by upregulated expression associated with susceptibility to infections such as influenza virus and West Nile virus (WNV) (45). Collectively, these findings suggest that inflammatory and chemokine-related transcriptional programs may be associated with the HS phenotype, although their causal contributions to COVID-19 susceptibility require validation in independent cohorts and functional studies.

Our study identifies an association between CCR5 and COVID-19 susceptibility (**Figure 1B**), suggesting that chemokine signaling may represent a common susceptibility mechanism across diverse pathogens. BTM analysis further showed higher enrichment of gene sets related to neutrophil recruitment and T-cell activation in HS individuals. Although neutrophils are essential components of innate immunity, excessive neutrophil recruitment has also been associated with inflammatory responses during viral infection (46, 47). WGCNA identified a T-cell activation-related module associated with the susceptibility phenotype (turquoise module; **Figures 2B–D**); This discrepancy may stem from functional polarization of T cell subsets (e.g., Th17/Treg imbalance) or inflammatory CD8+ T cell populations-mechanisms requiring validation via dynamic single-cell transcriptomics with TCR sequencing. Mirroring influenza studies, T cell heterogeneity (e.g., multifunctional IL-2+/IFNγ+ CD4+ vs. TNFα+ CD8+ cells) likely dictates infection outcomes, underscoring the need for subset-resolved analyses (10). Recent clinical studies have reported significantly altered neutrophil and lymphocyte responses in patients infected with Omicron variants, which is consistent with our findings (48). Collectively, these findings suggest that differences in neutrophil recruitment and T-cell activation may be associated with the HS phenotype, although their biological significance requires further experimental validation.

Vascular-related changes were also observed in the HS group. Angiogenesis-related pathways were enriched, and CDH5 expression was reduced in HS individuals (**Figure 1B**), suggesting possible differences in endothelial-related regulation (49). Because CDH5 encodes VE-cadherin, which is important for endothelial junction stability, this finding may be relevant to vascular responses during infection, although endothelial function was not directly examined in this study. SERPINE1 was also upregulated in the HS group (**Figure 1B**) (50). Lung organoid studies demonstrate SERPINE1-dependent viral proliferation, with inhibitors reducing viral titers in a dose-dependent manner (51). Notably, SERPINE1 expression correlates with HIF1A/VEGFA(**Figure 1B**) co-expression signatures, suggesting hypoxia-adaptive mechanisms that may modulate mucosal oxygen distribution and immune cell trafficking (52). However, these observations are based on transcriptomic associations and require further experimental validation.

Weighted gene co-expression network analysis further suggested an association between antigen-presentation-related transcriptional programs and the HS phenotype. The blue module was enriched in gene sets related to MHC class II binding and TLR signaling (blue module; **Figures 2C, D**), both of which are involved in antigen recognition and innate immune responses (50, 53). These modules were also associated with monocyte-related signatures, whereas GSEA showed lower enrichment of monocyte-associated gene sets in the HS group (**Supplementary Figure 5C**). This pattern may reflect differences in monocyte abundance or function, but these possibilities cannot be distinguished using bulk transcriptomic data. FURIN and PABPC4 were identified as hub genes in the turquoise module (**Figure 2F**). Previous studies have linked FURIN to SARS-CoV-2 spike processing and PABPC4 to coronavirus-related RNA regulation (54, 55). These findings support their potential relevance to the susceptibility-associated transcriptional phenotype, although their functional roles were not directly examined in this study and require further experimental validation.

IFN-related genes were identified in both the differential expression and WGCNA analyses. In the HS group, ssGSEA showed higher enrichment of IFN-related transcriptional signatures, whereas the expression of IFN receptor-associated genes, including IFNAR1 and IFNAR2, was lower (**Figure 3**). Type I IFN responses are known to be time-dependent, with early signaling contributing to viral control and delayed or insufficient responses being associated with ineffective antiviral defense and inflammation. Genomic studies have linked COVID-19 susceptibility to candidate genes in the type I IFN pathway, including IFNAR2 and IFNα-encoding genes (56). Clinically, severe patients display reduced serum IFNα levels and downregulated IFN-stimulated genes (ISGs), indicating impaired IFN signaling efficiency (57). Taken together, our results indicate differences in IFN-related transcriptional regulation between the HS and LS groups. However, IFN protein levels and signaling activity were not directly measured, and the functional significance of these differences requires further validation.

Several limitations of this study are noteworthy. Firstly, the volunteer population was mainly composed of college students from a specific region in China. This relatively homogeneous population may limit the generalizability of our findings to broader populations, including different age groups, ethnicities, and individuals with various underlying health conditions. Although age, sex, BMI, post-vaccination interval, and booster vaccination status were comparable between the HS and LS groups, residual confounding by unmeasured factors cannot be completely excluded. Secondly, because PBMC samples were collected following the after-lifting COVID-19 wave, residual post-infection or convalescent effects on gene expression cannot be fully excluded. However, longitudinal transcriptomic studies indicate that infection-induced peripheral blood changes in healthy individuals are largely reversible and tend to return toward baseline within weeks to months after recovery. In this study, COVID-19 susceptibility refers to the relatively stable host predisposition to develop clinically apparent infection under broadly comparable exposure, rather than the transient immune response induced during or immediately after infection. Considering the young, generally healthy cohort and predominantly mild or asymptomatic Omicron infections, such confounding may have been partially reduced, but future studies with pre-exposure baseline samples are needed for validation (23–25). Thirdly, COVID-19 susceptibility is multifactorial, and the binary HS/LS classification may oversimplify this complexity, particularly because the LS group includes both uninfected and asymptomatic individuals. Future studies with larger longitudinal cohorts and more refined multi-category stratification are needed to better define susceptibility-related immune mechanisms. Fourthly, the relatively small independent test set (n=35) may overestimate model performance. Thus, this model should be regarded as a preliminary exploratory framework, and larger independent cohorts and external independent validation dataset are needed to validate its generalizability and potential clinical utility.

Overall, our study provides an integrated framework for examining potential COVID-19 susceptibility-associated immune variation at the gene, network, and pathway levels, while offering a preliminary basis for future validation in prospective and independent cohorts.

## Data Availability

The raw sequencing data have been deposited in the National Genomics Data Center (NGDC) under accession number HRA011289 and are available at https://bigd.big.ac.cn/gsa-human/browse/HRA011289. The raw gene-level read-count matrix used for differential expression analysis, together with the corresponding de-identified sample-level clinical phenotype data, has also been submitted to the Open Archive for Miscellaneous Data (OMIX) under accession number OMIX019095.
