# Supplementary material for: Transcriptomic profiling reveals immune signatures associated with potential COVID-19 susceptibility and a predictive framework in a Chinese cohort
Source: Front Immunol. 2026 Aug 21;17:1847360. doi: 10.3389/fimmu.2026.1847360 (PMC13541666; doi:10.3389/fimmu.2026.1847360)
Supplement: Supplementary file 1 [file DataSheet1.docx]

# Supplementary material

**Supplementary Fig. 1** Integrated research design and analytical framework for infection susceptibility prediction.

**Supplementary Fig. 2** Visualization of the top 12 GO biological process terms enriched in DEGs. Each dot represents a term.

**Supplementary Fig. 3** PPI network of DEGs (STRING database v12.0, interaction score >0.4) Nodes colored by Log_2_FC (red: up, blue: down); edges represent experimental/curated interactions.

**Supplementary Fig. 4**. Co-expression network analysis by WGCNA. **a**, Sample clustering for outliers detection. **b**, Selection of the soft-thresholding powers. The x-axis is the soft-thresholding power and the y-axis is scale-free fit index. **c**, Mean connectivity across candidate soft-thresholding powers.

**Supplementary Fig. 5** GSEA analysis of the comparison between the HS and LS groups. **a**, curves of GSEA enrichment scores for enrichment in neutrophils. **b,** curves of GSEA enrichment scores for enrichment in T cells. **c,** curves of GSEA enrichment scores for enrichment in monocytes.

**Supplementary table. 1 hub genes in PPI network**

|  | genes |
| --- | --- |
| PPI | PDGFB,EGR2,FCGR3B,IL1B,H3C13,SERPINE1,CX3CR1,CALML4,GZMA,MMP1,H2AC8,CENPW,H2BC5,CCR5,CCNB1,H2BC15,CENPM,IFNG,H2BC4,TGFB2,CCN2,TNFSF10,EGR1,TNFSF11,PIK3R1,IL13,HIF1A,THBS1,SNAI1,CDH5 |

**Supplementary table. 2** hub genes in significant modules

|  | genes |
| --- | --- |
| blue | PSAP,APLP2,PLBD1,CD93,PLP2,TKT,GRB2,SCPEP1,ZDHHC7,CSTA,BST1,MAP3K3,CRTAP,HEXB,CHP1,TIMP2,GRK3,MACROH2A1 |
| turquoise | HSPA8,PABPC1,LBH,FOSL2,IFNGR1,SERINC1,DYNLT1,ELF1,RBM38,MOAP1,RBM3,FURIN,CCDC69,B4GALT1,HERPUD1,TAMALIN,PIK3R1,CBX4,RPA2,STK4,DYNLL1,FBXO33,ZBTB18,MRPL51,CD55,HNRNPUL1,AGFG1,JARID2,NCOA3,USF2,RNF5,KBTBD2,SEC24B,TMED4,ING4,CLINT1,HMOX2,KLF7,FCHSD2,WBP11,MAP4K4,TXN2,WDR74,COPZ1,MRPL24,WHAMM,ZC3H7A,PABPC4,YTHDF1,WDR43 |

**Supplementary table. 3** Model parameters

| Model | Parameters |
| --- | --- |
| RF(PPI) | RandomForestClassifier(max_depth=11,min_samples_split=18,n_estimators=58, random_state=42) |
| RF (WGCNA) | RandomForestClassifier(max_depth=14,min_samples_split=19,n_estimators=227,random_state=42) |
| LR (ssGSEA) | LogisticRegression(C=11.599167006256552,random_state=42) |
| Ensemble Model | LogisticRegression() |

**Supplementary table. 4** Model features

| Model | Features |
| --- | --- |
| RF (PPI) | PDGFB,H3C13,SERPINE1,CALML4,MMP1,CDH5,H2AC8,CENPW,H2BC5,CCNB1 |
| RF (WGCNA) | APLP2,GRB2,BST1,MAP3K3,CHP1,HSPA8,PABPC1,DYNLT1,ELF1,RBM38 |
| LR (ssGSEA) | CD79A,PPP3CA,IFNG,KRAS,CD40LG,CTSH,FCN1,PTPN6,F2RL2,FCGR2B |

**Supplementary table. 5**. Performance of different classifiers to predict COVID-19 susceptibility on the training dataset

| Classifier | Accuracy | Precision | Recall | F1 score | AUC |
| --- | --- | --- | --- | --- | --- |
| RF (PPI) | 0.88 | 0.90 | 0.79 | 0.84 | 0.97 |
| RF (WGCNA) | 0.89 | 0.89 | 0.84 | 0.87 | 0.96 |
| LR (ssGSEA) | 0.71 | 0.68 | 0.59 | 0.63 | 0.77 |
| Ensemble Model | 0.95 | 0.93 | 0.95 | 0.94 | 0.98 |
